# Institutional Notes and News

**Published:** 1911-03-18

**Authors:** 


### Institutional Notes and News.

**Change of Name in East London.**

By unanimous decision last week the name of the West Ham and East London Hospital has been changed to the West Ham and Eastern General Hospital. This change summarises in a phrase the progressive work done by the Institution, for a district which goes far beyond West Ham, beyond indeed East London, is now served by the hospital. This extended activity was reflected in two ways at the recent annual meeting, first by the enthusiasm which was then shown by the hospital's supporters, and secondly by the magnificent grant of £5,000 which King Edward's Hospital Fund has given in order to free the new buildings from debt. Thus the anxiety which was felt on this account so lately as December has been dispelled by the action of the Fund. The festival dinner in December proved a great success, realising £1,255, so a busy year had more than one success to encourage the hospital's authorities. The in-patients numbered 607, with the inevitably heavy proportion of cases to be attended in the out-patient department. The resignation of Miss Ough, the matron, has removed a very well-known figure, who had carried on her duties for a long period of years.

**School Children at the London Ophthalmic.**

We much regret having been compelled to hold over an account of the experiment undertaken by the Royal London Ophthalmic Hospital till this week. This hospital, the old "Moorfields Eye Hospital," is in a unique position, for it is practically the only hospital in London which has taken in hand the problem of the medical treatment of school children with any seriousness. Instead of confining itself to correspondence with the educational authority or to a protest against the new work, the Royal London Ophthalmic has started what is virtually a school clinic of its own. That is to say, the school children are segregated from the other out-patients and have a special department to themselves. The experience which this will give may not necessarily prove the final solution, but it will be invaluable to other institutions which in the future are practically certain in some way to co-operate with the educational officials. We shall watch the progress of this scheme with great interest, and shall report its developments as they occur. The hospital's subscribers ought to be encouraged by the initiative which has led to this public-spirited experiment.

**County Hospital, Liverpool.**

A very successful dinner in aid of the Royal Liverpool County Hospital for Children, quickly following on the annual meeting, has inaugurated this hospital's new year with great spirit. The peculiar work which it performs in meeting the needs of children who require long treatment, and in combining the effects of scientific open-air treatment with those of time, were noticed in our issue of November 12. It is instructive to compare that general statement of the County Hospital's aims with the facts and figures of the newly-issued annual report. There we find that "children treated in (the newly-built) open-air wards make more speedy recovery than was the case when they were placed even at night time only in closed structures," which allows more patients per bed in the year. In May the latest of these four wards, that dedicated to Mr. Holbrook Gaskell, will probably be open. The children treated last year numbered 158, and the above-mentioned dinner is the latest great effort made to impress upon Liverpool the value of the work which is being done. There is still a debt of £2,230 on the working account, but the brilliant gathering the other night, which included Lord Derby, the president, and Sir Thomas Barlow, Sir Donald Macalister, Mr. R. G. A. Moynihan, F.R.C.S., and the Lord Mayor, aroused a great deal of enthusiasm, and the speakers did all that speakers can do to impress on the public the value and the needs of this original and progressive hospital. Colonel R. Montgomery, V.D., received a most well-deserved recognition of the intense interest and enthusiasm which he has given as chairman to every new development of the hospital's work.

**Prince Christian at Windsor.**

Prince Christian, the president, took the chair at a most successful annual meeting a few days ago of the King Edward VII. Hospital, Windsor. There the Memorial Fund to the late King quickly rose to £11,000, and the fund has now been closed. The fund is to provide also for a statue of his late Majesty, by the Countess Feo Gleichen, which will be placed in front of the hospital. Everyone at Windsor, from Mr. Mayo Robson to the smallest subscriber, must be congratulated upon the success which has attended the Memorial Fund. Windsor now has paid a worthy tribute to the memory of King Edward, who honoured the Royal Borough by his periodic residence, and is now honoured by it in connection with the institution named already after him. Its maintenance will be the most useful local memorial of his work for hospitals in general.

**Hereford and the late King.**

The March monthly meeting of the Herefordshire General Hospital has left its chances of improvement precisely where they were in December last. Then, as we pointed out in our issue of the 10th instant, it was decided to support the hospital's claims at the proposed public meeting to be held for the consideration of the form of the county memorial. It now seems highly probable that Herefordshire will be known as the county with no memorial to King Edward. The public meeting proposed in December has not yet been held, and in March the only decision come to is to ask the Lord-Lieutenant of the county to consider "some scheme . . . . as a memorial to the late King." The four dots mark the omission of the all-important words "for the improvement of the hospital," which was finally decided on. This simply means that the hospital has been unable to resist some infection of the inertia which has prevailed in the county on the subject of commemorating King Edward's life and work. Indeed, struggling amid such astonishing indifference, it is praiseworthy to find the hospital to be the one place where the project is still discussed seriously. Lord Biddulph, who has consented to represent the hospital's claims for a new wing at the "proposed public meeting," should insist at least that this meeting be held, for it is monstrous that the people who are now objecting to the hospital's new wing as a memorial should be allowed to quash the project without being compelled to support another scheme of their own. We suspect that this other scheme (for a sanatorium) is talked of only in the hope that people will be frightened by it into doing nothing at all. We also agree with Mr. Child and Mr. Symonds-Taylor respectively in saying that it will be a disgrace to the county of Hereford if there is no memorial, and that if the hospital will only go to the public with a scheme, the public will provide the money. Nevertheless, the hospital must not allow the county to waste any more months of valuable time.

